# Supplementary material for: An immortalised mesenchymal stem cell line maintains mechano-responsive behaviour and can be used as a reporter of substrate stiffness
Source: Sci Rep. 2018 Jun 12;8:8981. doi: 10.1038/s41598-018-27346-9 (PMC5997644; doi:10.1038/s41598-018-27346-9)
Supplement: Supplementary file 1 — Supplemental Figures [file 41598_2018_27346_MOESM1_ESM.pdf]

Supplemental Figures

An immortalised mesenchymal stem cell line maintains mechano-responsive behaviour and can be used as a reporter of substrate stiffness

Asier Galarza Torre[1, 2, 4, 5], Joshua E. Shaw[1, 2, 4], Amber Wood[3, 4], Hamish T. J. Gilbert[1, 2, 4], Oana Dobre[1, 2, 4, 6], Paul Genever[7], Keith Brennan[3, 4], Stephen M. Richardson[2, 4] & Joe Swift[1, 2, 4] *

(1) Wellcome Centre for Cell-Matrix Research, University of Manchester, Manchester, M13 9PT, UK.

(2) Division of Cell Matrix Biology and Regenerative Medicine, School of Biological Sciences, University of Manchester, Manchester, UK.

(3) Division of Cancer Sciences, School of Medical Sciences, University of Manchester, Manchester, UK.

(4) Faculty of Biology, Medicine and Health, University of Manchester, Manchester Academic Health Science Centre, Manchester, M13 9PL, UK.

(5) Present address: Scottish Centre for Regenerative Medicine, Edinburgh, EH16 4UU, UK.

(6) Present address: School of Engineering, University of Glasgow, Glasgow, G12 8QQ, UK.

(7) Department of Biology, University of York, York, YO10 5DD, UK.

* Corresponding: joe.swift@manchester.ac.uk

Fig. S1.

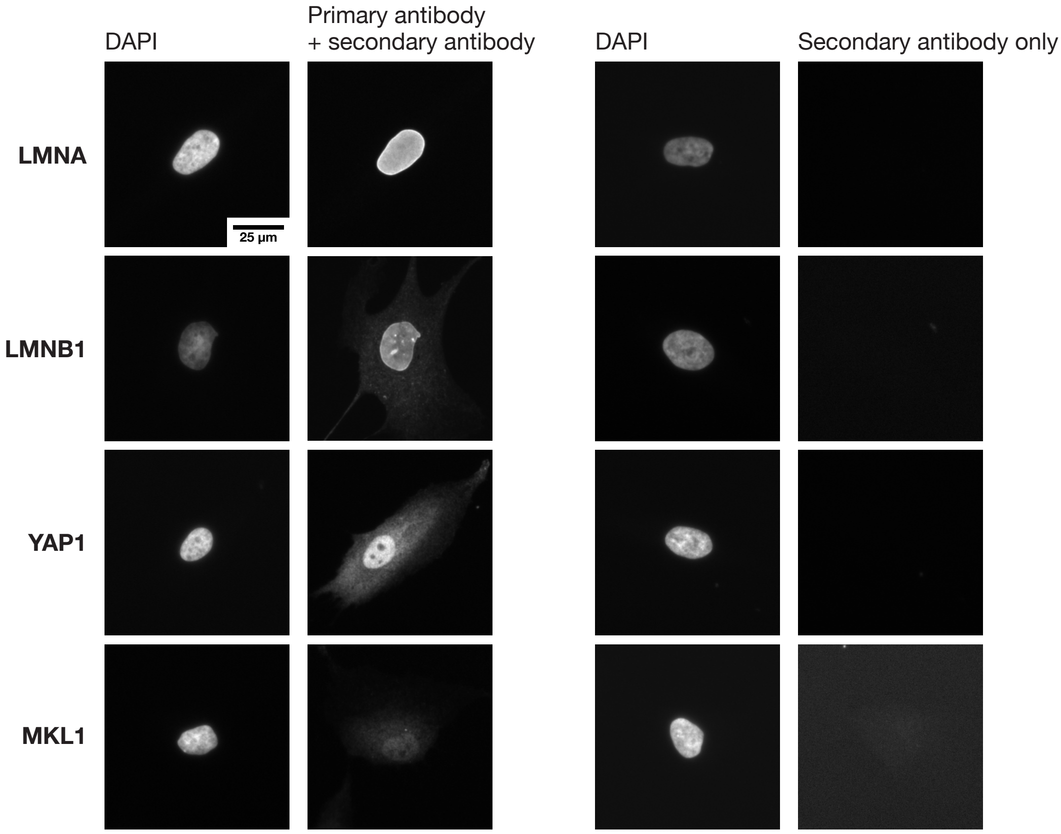

Supplemental Figure 1. Secondary-only controls for antibody staining experiments. Immortalised human MSCs were imaged with and without primary antibody staining. Cells were imaged under the same conditions and microscope settings.

Fig. S2.

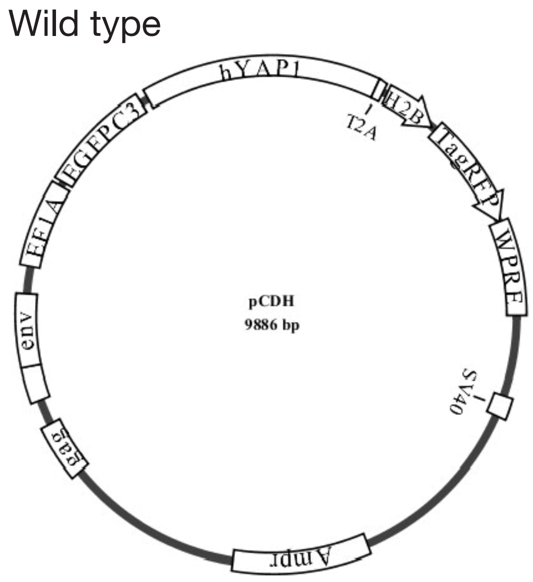

Supplemental Figure 2. Plasmid maps of constructs used to create reporter cells. Lentiviral backbone plasmids contained the human YAP1 gene tagged to GFP linked with the T2A peptide to histone H2B tagged to RFP. This bicistronic vector allowed for expression of both proteins in the same reading frame, in a stoichiometric manner. Transcription was regulated by an EF1A promoter. The 4SA plasmid has four point mutations on YAP1 serine residues: S61A, S109A, S164A and S381G.
